# A Smart Phone Based Handheld Wireless Spirometer with Functions and Precision Comparable to Laboratory Spirometers

**DOI:** 10.3390/s19112487

**Published:** 2019-05-31

**Authors:** Ping Zhou, Liu Yang, Yao-Xiong Huang

**Affiliations:** Department of Biomedical Engineering, Ji Nan University, Guang Zhou 510632, China; tmyield@jnu.edu.cn (P.Z.); lyang246-c@my.cityu.edu.hk (L.Y.)

**Keywords:** Portable spirometer, Lilly flowhead, smart phone base, handheld unit

## Abstract

We report a smart phone based handheld wireless spirometer which uses a Lilly type sensing flowhead for respiratory signal acquisition and transmits the data to smartphone or other mobile terminals with Bluetooth signal transmission for data processing and result display. The developed spirometer was demonstrated to be able to detect flow rates ranging from 0–15 L/s with an accuracy of 4 mL/s, and can perform tests of flow volume (FV), forced vital capacity (FVC), forced expiratory volume in 1 s (FEV1), peak expiratory flow (PEF), etc. By having the functions and precision comparable to laboratory spirometers, it satisfies the American Thoracic Society and European Respiratory Society (ATS/ERS) proposed performance requirements for spirometer. At the same time, it is low cost, light and handy, low power consumption battery-powered. The test of 12 cases of subjects using the developed spirometer also indicated that it was easy to use for both providers and patients, and suitable for the Point of Care Test (POCT) of chronic obstructive pulmonary disease (COPD) and asthma at general-practice settings and homes.

## 1. Introduction

Millions people in the world are suffering from chronic obstructive pulmonary disease (COPD) and asthma [1], the two most common respiratory diseases which can seriously impact people’s health, and even cause death. Patients with asthma and COPD are recommended to perform daily measurements of static and dynamic respiratory functions to track their pulmonary health with ease and to prevent exacerbations [2] Spirometers are the most common machines for such tests [3,4]. They measure the volume and speed of air that is inhaled and exhaled to assess lung function and to provide a first-level diagnostic test for the pulmonary diseases and the progression of disease. 

There are two basic classes of spirometers: laboratory units and portable ones [5,6]. Laboratory spirometers are either desktop consoles or cabinet-size machines usually placed in hospitals and operated by trained technicians [7]. Though they can provide precise spirometry measurements and perform systematic tests on flow volume, tidal spirometry, and maximum voluntary ventilation, it is inconvenient and uneconomic for patients to go to hospital daily for the test. Since laboratory spirometers are expensive, their use by primary care providers is not widespread. Portable spirometers are usually either compact desktop units or handheld devices for general-practice and home use [6,8]. They are small and their power consumption is low. Low cost is also an important characteristic feature of these kinds of spirometers, so they are more accessible to a greater population of users. 

At their early stage, portable or handheld spirometer mainly took the forms of Wright and turbine peak flow meters [9,10]. Most of them were just able to perform simple peak expiratory flow test, and have low accuracy owing to the momentum and resistance errors associated with the moving parts such as windmills or flow valves in these kinds of equipment. Furthermore, they did not have adequate functions and lacked capabilities to display the graphical information such as volume-time and flow-volume graphs [3,4,6,11]. Though later on, some of them had become digitalized and some other kinds of spirometers based on the methods of differential pressure [12], ultrasound [13], and hot-wire [14] flowmeters had also developed. The improved spirometers still could not perform systematic spirometry tests and give graphical information without connecting to a PC or a console. Recently, by the aid of the technical development of wireless communication and smartphone/iPad, some wireless and smartphone/iPad based portable spirometers have been developed to include functions of wireless communication and data calculation as well as graphic display [15,16]. However, most of them did not have accuracy comparable to laboratory spirometers [15,16,17], and therefore did not meet the requirement published by the American Thoracic Society and European Respiratory Society (ATS/ERS) for the flow range of 0–14 L/s and minimum detectable flow of 25 mL/s [18,19]. Moreover, they are not low cost, and usually cost over $1000. Therefore, there is a great need for a portable/handheld spirometer which is light, portable and low cost, but is also able to perform precise and systematic spirometry measurements, with functions comparable to laboratory spirometers. At the same time, it should wirelessly transmit test results to family doctor/hospital for guidance. Based on our knowledge, none of the previously reported portable spirometers were able to meet all these demands. The purpose of the present paper is to outline the development of such a spirometer with innovations in flow sensing unit design, high fidelity signal acquisition and data processing, and of which operation is based on a user-friendly operation application (app) for smartphones or other mobile terminals.

## 2. System Design

### 2.1. General Consideration

To be light and portable, our spirometer should be a wireless small compact handheld unit without any external connecting pipes/tubes and desktop consoles. It should have functions and precision comparable to laboratory spirometers, so as to perform a range of tests, such as flow volume (FV), forced vital capacity (FVC), forced expiratory volume in 1 s (FEV1), peak expiratory flow (PEF), maximum voluntary ventilation, and tidal spirometry, etc. It should also meet the performance index requirements for spirometer recommended by the American Thoracic Society/European Respiratory Society (ATS/ERS) [18,19]. Specifically, it should be able to respond flow rates ranging from 0 to 14 L/s with a resolution better than 25 mL/s, and have a precision of ≤±3% for FVC, and ±10% for PEF. Moreover, it should have the capacity to display the test results including both data and graphics immediately after the test, and at the same time transmit them to family doctors or a hospital unit. To solve these problems, considering the recent popularity of smartphones, we designed the spirometer as a smartphone based one so that it just consists of a small handheld Lilly type flowhead, which has the capability to communicate with a smartphone wirelessly. In this way, the data processing and result displaying are performed by the smartphone of the user, So that the structure of the spirometer is simplified, and its size and cost are also reduced.

**Figure 1:**
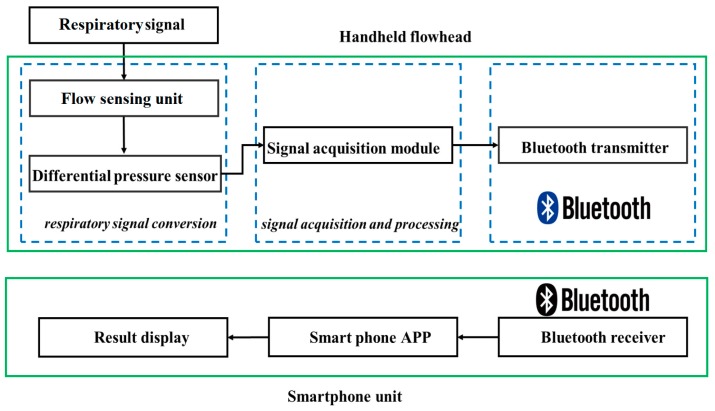
The diagram of the handheld wireless spirometer.

Our system consists of five parts: a flow sensing unit composed with a Lilly type pneumotachometer and a high-precision differential pressure sensor; a signal conversion and acquisition module based on a mixed-signal processor; a Bluetooth signal transmission module; a mobile terminal with wireless signal receiver, processor, display module and app; and a rechargeable lithium battery power supply circuit. Figure 1 shows a diagram of the spirometer.

### 2.2. Handheld Flowhead

#### 2.2.1. Lilly Type Flow Sensing Unit

**Figure 2:**
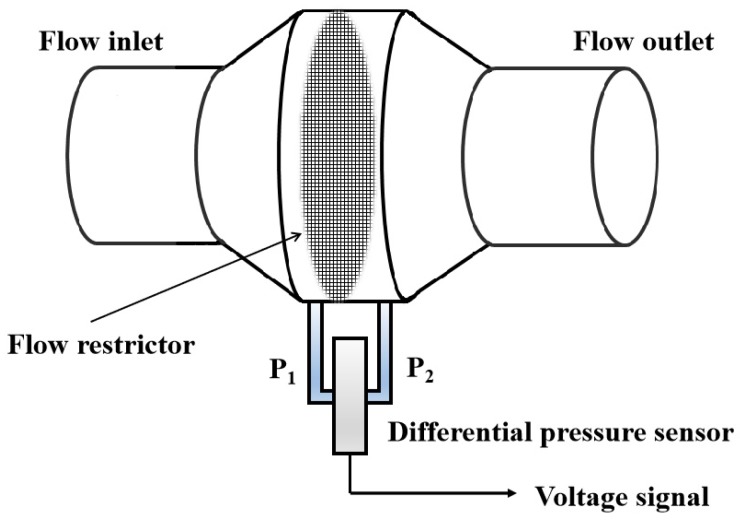
The structure of Lilly type sensing unit.

The Lilly type sensing unit uses a flow restrictor to create a linear flow/differential pressure relationship at both sides of the restrictor immediately when the air flow travels through it (see Figure 2) according to the Poiseuille’s law:

Δ*P* = *Q R*. (1)

Here, *Q* is the flow rate, *R* is the flow resistance, Δ*P* is the pressure difference. Its performance is well understood, and in comparison with other types of flowmeters such as turbine [10], Hot-wire [14], ultrasonic [13], Pitot tube [20] and Venturi tube types [12], it is simpler to make and cheaper, containing no moving parts but with a satisfactory accuracy. 

#### 2.2.2. Differential Pressure Sensor

The sensor is a SDP1000-L differential pressure sensor manufactured by Swiss Sensirion Company. The reason this kind of sensor was chosen instead of a digital one is that it is low cost, smaller in size, wider responding pressure range while with high precision in converting the pressure difference created by the Lilly type sensing unit into analog voltage with linear calibration and temperature compensation. It gives out 0.25 V–4 V electrical potential corresponding to the pressure ranging from 0–500 Pa with a precision of 1 mV, so it can fully meet the requirements about the testing range (0–14 L/s) and resolution (≤25 mL/s) of our spirometer. 

#### 2.2.3. Signal Conversion and Acquisition Module

**Figure 3:**
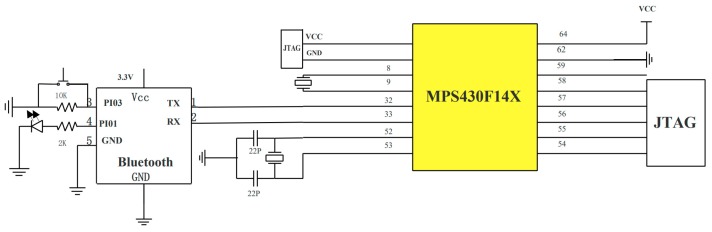
The connections and circuits of MSP430F149.

The module composes of a MSP430F149 (TI company, Dallas, TX, USA) single-chip microcomputer which is of 60 KB+256 bit FLASH and a 16-bit mixed-signal processor with 12-bit ADC (Analog-to-Digital Converting) function. The mixed signal processor MSP430 receives the analog voltage signal from a SDP1000-L differential pressure sensor and converts them into a digital signal with the 12-bit ADC. The frequency of the signal acquisition was provided by the peripheral 32,768 Hz and the sampling frequency can be set to 100–200 Hz, according to requirements. The connections and circuits of MSP430F149 are shown in Figure 3.

#### 2.2.4. Rechargeable Lithium Battery Power Supply

**Figure 4:**
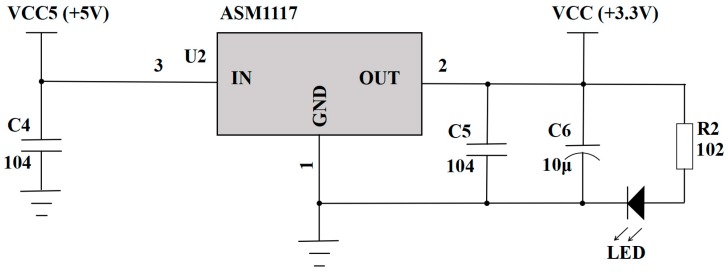
The level conversion circuit of the power supply.

The 5 V constant voltage source is powered by a rechargeable lithium battery. A level conversion circuit (see Figure 4) is used to realize a 5 V to 3.3 V conversion for the power supplies of differential pressure sensor and the MSP430 module.

#### 2.2.5. Bluetooth Wireless Transmission 

The Bluetooth wireless transmission uses two HS-05-USB modules of Huicheng Company (Guang Zhou, China). In the handheld flowhead, it serves as the slave while in the mobile terminal, it is the host. After powering the mixed signal processor and Bluetooth, the host searches for a slave and establishes a communication relationship when the password is matched. The collected data is then converted to Bluetooth communication format and sent to the cache of the Bluetooth host at a rate of 100 6-byte data packets per second. 

### 2.3. Mobile Terminal 

The mobile terminal can be a smartphone or a tablet, etc. It should have a wireless signal receiver, a processor and displaying module accompanied with a specially designed app for spirometry. It receives the Bluetooth data and reads them with the app. After each data packet is identified, it extracts the digital quantity and then processes them to restore the respiratory signal. The displaying module displays patient information, the spirometry parameters, spirograms (usually exhibited as flow-time curves and volume-time curves), and system information.

### 2.4. Operation App

**Figure 5:**
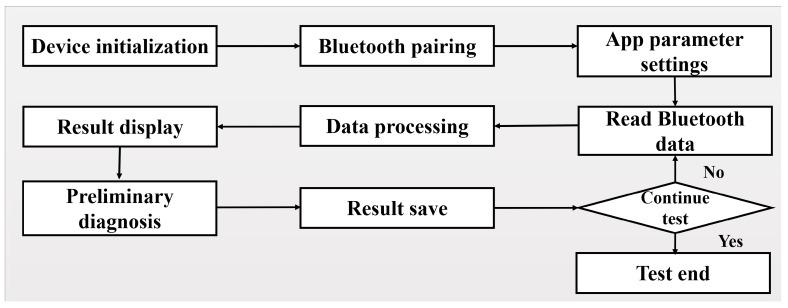
The flowchart of the spirometry app.

The app is responsible for: the operation of the spirometry test, including the initialization of the Bluetooth device to pre-configure the communication mode, searching for the Bluetooth device, and completing the pairing of the Bluetooth; setting the app parameters; reading data from the Bluetooth receive buffer; data processing and the result (including both data and graphic) display. Data processing includes extracting lung function data according to the transmitted packet pattern and calculating the data according to a designed algorithm. The app also sends patient information and performs communication with the doctor and hospital unit about the test results and their consultation and guidance. The flowchart of the app is shown in Figure 5.

## 3. Results and Discussion

**Figure 6:**
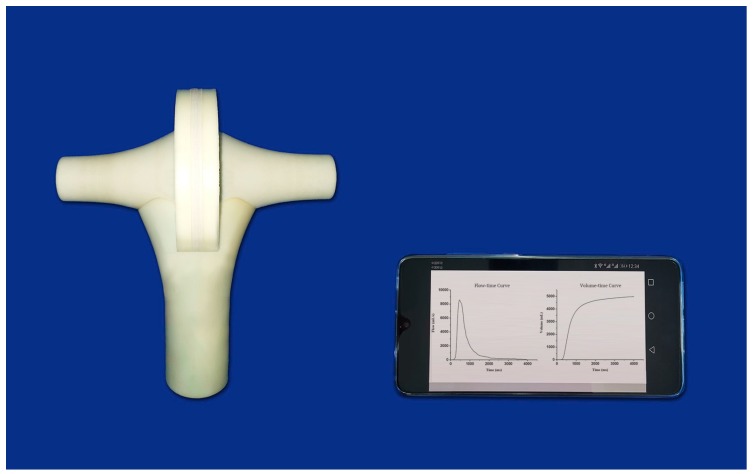
The handheld wireless flowhead and the smartphone displaying the results of the spirometry.

Apart from the differential pressure sensor, we integrated all the other electronic parts, including the signal conversion and acquisition module, the Bluetooth wireless transmission circuit and the power supply circuit into a small printed circuit board, so that the flowhead can be compacted to a small handheld unit as shown in Figure 6. 

**Figure 7:**
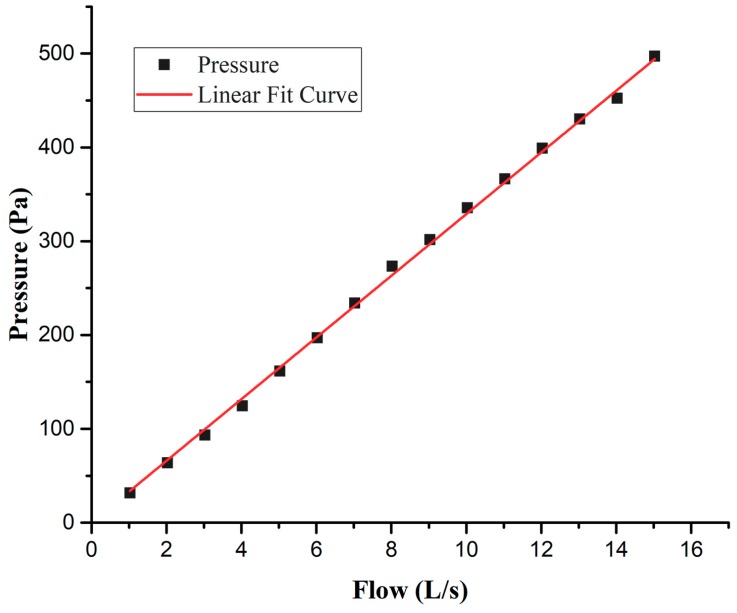
The resulting curve obtained from the test about the relation between flow rate and pressure difference of the handheld Lilly type sensing unit.

Considering that the range of the testing flow recommended by ATS/ERS is 0–14 L/s and the responding range of SDP1000-L differential pressure sensor is 0–500 Pa, we used a restrictor with flow resistance *R* ≈ 3.3 × 10^4^ Pa∙S/m^3^ so that the Lilly flow sensing unit has excellent linearity for the flow/pressure difference relationship, well within the responding range of the differential pressure sensor. Figure 7 is the resulting curve, obtained from the test about the relation between flow rate and pressure difference using a Thor Medical Systems-Pulmonary Waveform Generator (Stuhlweissenburg, Germany) as the flow generator. The correlation coefficient R^2^ = 0.997 indicates that the linearity of the flow/differential pressure relationship is satisfied. The test also suggested that the developed spirometer can detect flow rate from 0 to 15 L/s. As previously mentioned, the differential pressure sensor SDP1000-L gives out 0.25 V–4 V potential difference, corresponding to the pressure ranging from 0–500 Pa with a precision of 1 mV. The 1 mV electrical potential corresponds to a pressure difference of 0.133 Pa, or a flow rate of 4 mL/s according to the relationship between pressure and flow rate given in Figure 7. This was also demonstrated in the test performed to obtain the pressure-flow curve shown in Figure 7, with the Waveform Generator following the guidance of ATS/ERS for spirometer accuracy evaluation. Therefore, both the responding range of flow rate and the resolution of our spirometer are better than the requirements recommended by ATS/ERS (flow rate: 0–14 L/s, resolution: ±25 mL/s).

**Figure 8:**
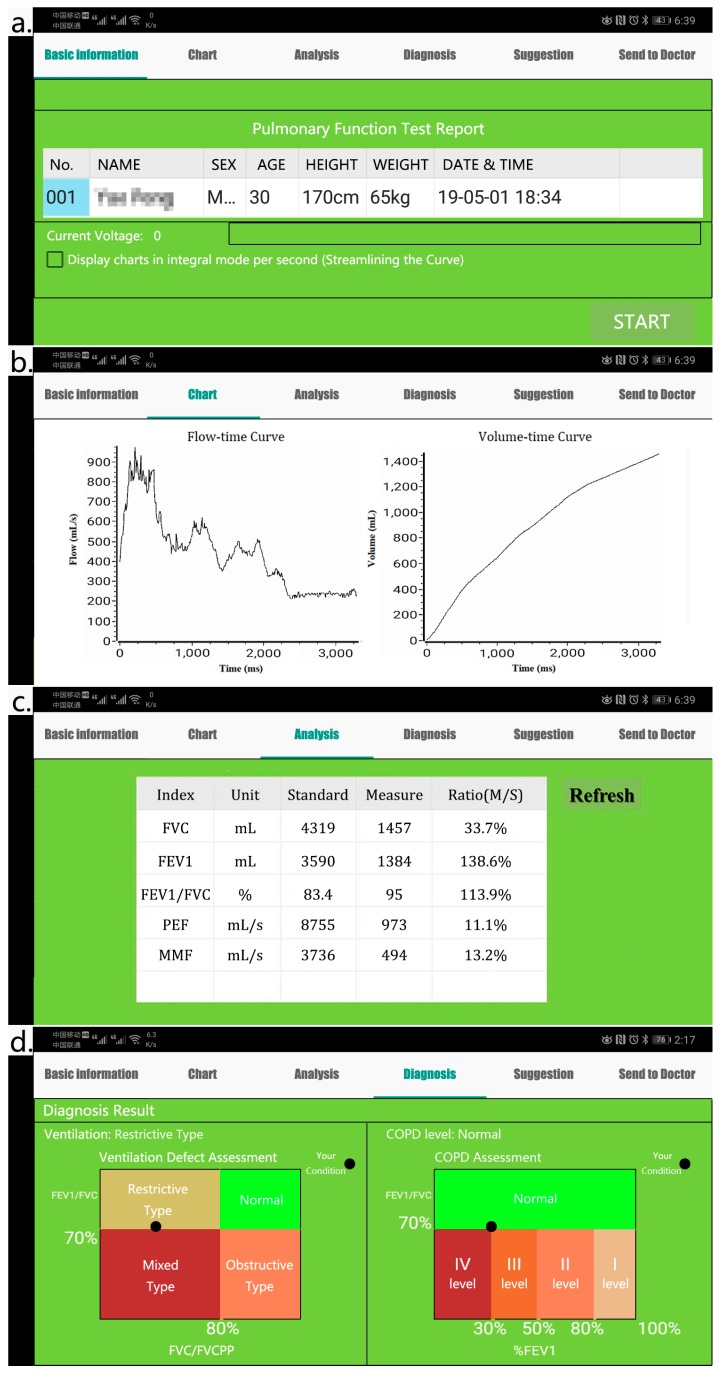
The displays of the smartphone app following a patient’s use of the developed spirometry test system. (**a**) the basic information of the person; (**b**) the flow-time curve and volume-time curve of the test, (**c**) the detailed data of FVC, FEV1, FEV1/ FVC, PEF and MMF; (**d**) the ventilation defect and COPD level assessments of the person.

Figure 8 shows several smartphone app displays of a patient who used the developed system to perform a spirometry test, which includes the basic information of the person (Figure 8a), the flow-time curve and volume-time curve of the test (Figure 8b), the detailed data of FVC, FEV1, FEV1/ FVC, PEF and MMF (Figure 8c), as well as the ventilation defect and COPD level assessments of the person, produced by the app (Figure 8d). The users can also easily use the app to send the results to the patient’s family doctor or hospital for consultation and guidance, or save the test data and results to track the variation of his/her condition, and add new tests. 

**Figure 9:**
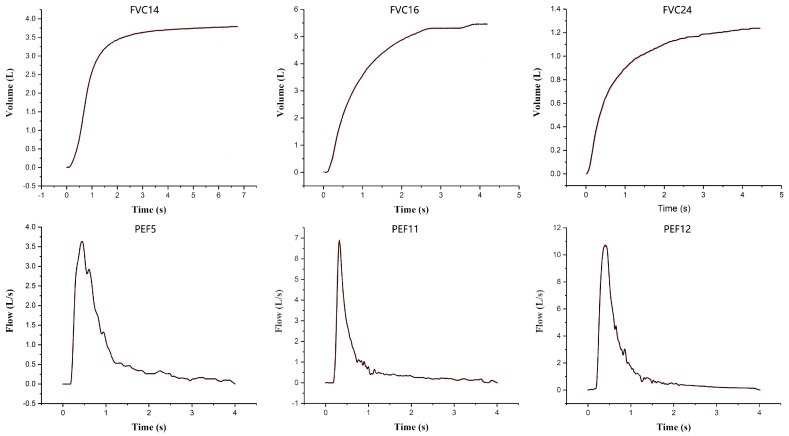
The resulting curves (the black curves) of three FVC and three PEF waveform tests using the developed spirometer and the standard ones (the red curves).

To test the validity, accuracy and reliability of our system, we used a Thor Medical Systems-Pulmonary Waveform Generator to create three standard ATS/ERS FVC waveforms (No. 14, 16 and 24) and three PEF waveforms (No. 5, 11 and 12) for the performance test of the developed spirometer in detecting pulmonary functions. Each of the waveforms was tested three to five times. Since the selected waveforms are the typical ATS/ERS standard waveforms, and include both extremely high (PEF 12) and low (PEF 5) flow rates, and different waveform shapes (rise time), such as rapid acceleration followed by rapid deceleration (PEF 11) or slow deceleration (PEF 5), they are capable of fully testing the performance of our pulmonary function test system. We can see from Figure 9 that all the measured waveforms were highly consistent with the standard ones; the standard error between the measured waveforms and the standard ones for the FVC test were all within 3%, and those for the PEF test were within 6% (see Table 1). The relative standard deviation (RSD) of the measurements for each test was less than 4%. Since these kinds of tests were the standard performance tests recommended by ATS/ERS and performed under the ATS/ERS spirometry guidelines, the results suggest that the developed spirometer has high accuracy and reliability (repeatability) in pulmonary function tests. 

In Table 1, Error (rate) was calculated by the following formula:

(2) δ=∑(Mk−Sk)SkN×100%

where *M_k_* is the value measured by the spirometer at point *k*, *S_k_* is the standard value generated by the Waveform Generator, the range of *k* are from 1 − *N*, *N* is the total number of measured points which were measured every 2 ms at the FVC and PEF curves. And:

(3) $$RSD=\frac{S}{\bar{x}}\times 100\%=\frac{\sqrt{\frac{\sum_{i=1}^{n}{\left(x_{i}-\bar{x}\right)}^{2}}{n-1}}}{\bar{x}}\times 100\%$$

Here *S* is the standard deviation, *x* is the measured value.

The practical usability of the developed spirometer was also evaluated by testing with 12 subject cases (8 males and 4 females, aged from 20 to 65 years). All of the tested persons performed the spirometry test by themselves either at home or at our lab. The persons also performed a spirometry test with a standard laboratory spirometer (Australia AD Instruments PowerLab). All the subjects were tested three times with each of the spirometers. Table 2 gives the test results of the subjects with our system (denoted as DS) and the AD instrument. We can see that the data obtained from both the systems were quite consistent. All the users had smooth operation of spirometry test without hindrance and claimed that the developed spirometer was easy to use, indicating that the developed spirometer is of comparable function and accuracy to a laboratory spirometer, and suitable for home monitoring.

The developed spirometer is a low power consumption device. It just needs 40 mA on average to conduct a 20 s whole set test including respiration signal acquisition and data transmission to the mobile terminal. So, for the 300 mAh battery employed in our spirometer, it can be used for 1350 tests. This means that even performing 10 tests each day, the developed spirometer can continually operate for 5 weeks without the need to recharge the battery.

Table 3 lists the specifications, principle of measurement, size and weight, the means of result display and the cost of some commercial portable spirometers that compare with our system. It indicates that the advantages of our developed spirometer over other major commercially available portable spirometers are that there is better resolution and accuracy, with much lower costs. There are also some pressure-based spirometers such as NeuLog® Spirometer Sensor [21], however, these kinds of spirometers need to connect the flowhead with the sensor by tubes/pipes, and work in a flow range ≤10 L/s with poor resolution (200 mL/s) [21]. Therefore, we have not listed them in Table 3 for comparison.

## 4. Conclusion

We have developed a smart phone (or other mobile terminals) based handheld wireless spirometer which is a light and portable wireless handheld unit. It consists of just a Lilly type sensing flowhead with Bluetooth wireless transmitter for respiratory signal acquisition and data wireless transmission, while the data processing and result displaying are completed by a smart phone or other mobile terminals. According to the experimental results, the developed spirometer can detect flow rates ranging from 0–15 L/s with a resolution and accuracy of 4 mL/s, and can perform tests of flow volume (FV), forced vital capacity (FVC), forced expiratory volume in 1s (FEV1), peak expiratory flow (PEF), etc. So, it has the functions and precision comparable to laboratory spirometers and satisfies the performance index requirements proposed by ATS/ERS for spirometer, as well as being low cost, portable, low power consumption, battery-powered, and is easy to use for both health providers and patients. In comparison with other recently available portable spirometers in the world, its advantages are that it has better resolution and test accuracy, is lower cost, and meets all the demands of an innovative portable spirometer mentioned in the introduction section. Therefore, it is expected to be popular, and to enable the POCT of COPD and asthma to shift from clinical laboratories to general-practice settings and homes. It will not only enable the patients of COPD and asthma to monitor the health of their lungs at home more effectively and more frequently, but also increase their communications with healthcare providers about their test results over the phone or the internet, to obtain timely consultation and guidance.

## Figures and Tables

**Table 1:** The accuracy and reliability of the developed spirometer in pulmonary function tests.

Waveform	FVC14	FVC16	FVC24	PEF5	PEF11	PEF12
Error	1.70%	−2.60%	1.30%	−2.80%	−3.90%	5.80%
RSD	1.22%	1.83%	1.37%	2.71%	2.85%	3.66%

**Table 2:** Test results of 12 subjects with two spirometers.

Person	Device	FVC (L)	FEV1 (L)	PEF (L/s)	MMF (L/s)	MEF75	MEF25
1	DS	3.89 ± 0.21	3.25 ± 0.09	4.99 ± 0.93	2.53 ± 0.59	4.58 ± 0.79	2.19 ± 0.13
	AD	3.90 ± 0.13	3.14 ± 0.29	4.69 ± 1.17	2.34 ± 0.66	4.42 ± 1.21	2.10 ± 0.12
2	DS	4.38 ± 0.11	3.58 ± 0.21	7.98 ± 0.97	3.56 ± 0.75	7.15 ± 0.93	1.64 ± 0.14
	AD	4.33 ± 0.27	3.65 ± 0.16	7.94 ± 1.32	3.47 ± 0.59	7.22 ± 0.41	1.68 ± 0.09
3	DS	3.74 ± 0.09	2.79 ± 0.07	3.23 ± 2.51	2.44 ± 0.38	3.12 ± 1.28	2.37 ± 0.05
	AD	3.68 ± 0.13	2.78 ± 0.13	3.07 ± 0.66	2.85 ± 0.56	2.91 ± 0.67	2.41 ± 0.12
4	DS	4.07 ± 0.10	3.42 ± 0.13	4.89 ± 0.96	2.71 ± 0.74	4.27 ± 1.09	2.25 ± 0.14
	AD	3.91 ± 0.18	3.24 ± 0.24	4.18 ± 1.37	2.52 ± 0.88	4.07 ± 0.54	2.39 ± 0.07
5	DS	5.30 ± 0.17	4.67 ± 0.21	6.74 ± 0.89	5.01 ± 0.93	6.51 ± 0.95	3.62 ± 0.07
	AD	5.38 ± 0.09	4.78 ± 0.11	6.56 ± 0.67	4.95 ± 0.67	6.02 ± 0.84	3.68 ± 0.09
6	DS	3.90 ± 0.25	3.24 ± 0.15	4.89 ± 0.48	2.30 ± 0.50	4.08 ± 0.78	2.16 ± 0.10
	AD	3.91 ± 0.14	3.34 ± 0.06	4.87 ± 0.55	3.70 ± 0.81	4.56 ± 1.19	2.24 ± 0.08
7	DS	2.54 ± 0.17	2.54 ± 0.04	5.16 ± 1.78	3.95 ± 0.58	4.77 ± 1.14	2.46 ± 0.16
	AD	2.42 ± 0.26	2.77 ± 0.28	4.71 ± 1.59	4.03 ± 0.86	4.60 ± 0.63	2.21 ± 0.12
8	DS	4.00 ± 0.05	3.41 ± 0.16	4.94 ± 1.28	3.72 ± 0.77	4.83 ± 0.98	2.35 ± 0.13
	AD	4.00 ± 0.22	3.47 ± 0.18	5.02 ± 1.41	3.99 ± 0.61	5.65 ± 1.27	2.18 ± 0.16
9	DS	3.30 ± 0.24	3.28 ± 0.11	7.56 ± 1.12	5.32 ± 0.79	7.10 ± 0.91	4.60 ± 0.06
	AD	3.33 ± 0.12	3.24 ± 0.14	7.88 ± 0.89	5.10 ± 0.96	7.68 ± 0.87	4.47 ± 0.14
10	DS	3.80 ± 0.13	3.05 ± 0.21	4.89 ± 1.48	3.15 ± 0.97	3.77 ± 0.46	2.21 ± 0.08
	AD	3.93 ± 0.25	3.38 ± 0.17	5.51 ± 0.94	3.91 ± 0.55	4.35 ± 1.72	2.19 ± 0.08
11	DS	3.13 ± 0.10	3.12 ± 0.14	6.44 ± 1.33	5.36 ± 0.75	6.30 ± 0.91	4.25 ± 0.13
	AD	2.93 ± 0.16	2.90 ± 0.23	6.34 ± 1.21	5.01 ± 0.66	6.16 ± 0.85	4.20 ± 0.09
12	DS	4.16 ± 0.18	3.43 ± 0.19	5.46 ± 0.73	3.82 ± 0.78	4.85 ± 0.56	2.93 ± 0.18
	AD	4.03 ± 0.27	4.03 ± 0.25	5.38 ± 0.89	3.94 ± 0.54	5.13 ± 0.67	3.12 ± 0.07

**Table 3:** Comparison of the developed spirometer with commercial ones.

	MIR Spirobank II	SDI Diagnostics Astra 100	SpiroTube	GoSpio	Ours
Flow range and resolution	0–16 L/s	0–16 L/s	0–18 L/s	0–14 L/s	0–15 L/s
	200 mL/s	150 mL/s	8 mL/s	25 mL/s	4 mL/s
Working principle	Compact turbine	Bi-directional digital turbine	ultrasonic multiple-path	Bi-directional vertical turbine	Differential pressure
Size and weight	160 × 55 × 25	147 × 83 × 102	150 × 60 × 21	120 × 100 × 80	120 × 100 × 70
	145 g	250 g	141 g	300 g	260 g
Based on PC or smartphone	PC, iPad	PC	PC	PC or smartphone	Smartphone or PC/iPad
Wireless or not	Yes	Not	Yes	Yes	Yes
Cost	$795	$1978	$1950.95	$1395	~$100

* Detailed information of MIR Spirobank II [15], SDI Diagnostics Astra 100 [17], SpiroTube [22] and GoSpio [16] can be found in the cited references.
